# Recanalizing distal and small medium vessel occlusions – single-center experience with the new Q catheters

**DOI:** 10.1007/s00234-026-04026-4

**Published:** 2026-05-09

**Authors:** Michael Griessmair, Christian Maegerlein, Dominik Sepp, Dennis Martin Hedderich, Jannis Bodden, Silke Wunderlich, Claus Zimmer, Jan Kirschke, Maria Berndt-Mück, Martin Renz, Tobias Boeckh-Behrens

**Affiliations:** 1 Department of Neuroradiology, Technical University of Munich, Munich, Germany; 2 Department of Diagnostic, Interventional and Pediatric Radiology, University Hospital of Bern, Bern, Switzerland; 3 Department of Neurology, Technical University of Munich, Munich, Germany

**Keywords:** MeVO (medium vessel occlusion), DVO (distal vessel occlusion), TICI (treatment in cerebral ischemia)

## Abstract

**Background:**

Mechanical recanalization of distal and small medium vessel occlusions (DVOs and MeVOs) is technically challenging and likely associated with higher complication rates. Previous studies have shown promising results using Q aspiration catheters in MeVOs. We report our experience with Q catheters in an even more distal cohort of DVOs and small MeVOs, focusing on safety and efficacy.

**Methods:**

We retrospectively reviewed our institutional stroke database for patients with DVOs or MeVOs treated using Q catheters. Baseline characteristics, technical outcomes including modified Thrombolysis in Cerebral Infarction (mTICI) score and modified first pass effect (mFPE), as well as clinical outcomes measured by the 90-day modified Rankin Scale (mRS) were analyzed. Outcomes were stratified by occlusion type and treatment technique. Periprocedural complications and their clinical impact were systematically assessed.

**Results:**

57 occlusions in 47 patients were included. Mean age was 75.5 ± 12.7 years. Median National Institutes of Health Stroke Scale score was 7.7 ± 4.6 at admission and 4.9 ± 5.5 at discharge, while the rate of 90-day- mRS ≤ 2 was 37%. 39 were DVOs and 18 small MeVOs. Successful recanalization (mTICI ≥2b) was achieved in 75% overall, with lower rates in DVOs (71%) than MeVOs (83%). Stent retriever–assisted aspiration resulted in higher recanalization rates than aspiration alone (82% vs. 37%). mFPE was achieved in 42%. Periprocedural complications occurred in 9% of cases with no symptomatic intracranial hemorrhage (sICH) or PH2.

**Conclusion:**

Q catheters appear to be safe and effective for DVOs and small MeVOs, achieving favorable recanalization rates with an acceptable complication profile. While the study is limited by its small sample size and retrospective design, these findings support the potential of Q catheters as a valuable tool in the treatment of distal and small vessel occlusions. Nevertheless, consistent to the recently published large distal trials, recanalization rates are lower, and complications occur more often in DVOs than in MeVOs, reflecting a different balance between the technical challenges and the smaller expected clinical benefit.

**Supplementary Information:**

The online version contains supplementary material available at https://doi.org/10.1007/s00234-026-04026-4.

## Introduction

After mechanical thrombectomy, particularly in LVOs (Large Vessel Occlusions), has become firmly established in stroke therapy in recent years several [1–3] new frontiers for the mechanical approach have been opened up, e.g. extending the time window [4–6], and, especially, treating smaller and more distal vessels, where three randomized trials have recently been presented and two of them being published [7–9]. However, in these trials, the actual size of the treated MeVOs (Medium Vessel Occlusion) and DVOs (Distal Vessel Occlusion) has not been taken into account as well as the technical details of the recanalization procedures.

Obvious obstacles in the interventional treatment of smaller MeVOs and DVOs are the increasingly challenging navigation in the distal vasculature and the structural fragility of those vessels. The resulting increased complication risk in addition to probably lower success rates and a smaller clinical benefit to be expected in less affected brain tissue may lower the risk-benefit ratio [10]. Therefore, besides creating clinical evidence for these occlusions, evaluations of adapted and suited recanalization tools and techniques are crucial.

There is, in addition to some case studies, one multicenter series evaluating the novel MIVI Q aspiration catheters– designed with a proximal 0.020-inch pusher wire and 0.088-inch guide catheter compatibility to significantly enhance aspiration force – in standard MeVO recanalization with pure aspiration [11], showing high success with low complications.

Additionally, to the recently published RCTs, one larger analysis of distal occlusions in the STAR registry is published to date [12]. However, data on smaller MeVOs and DVOs, and on the use of Q catheters in these locations, are still lacking.

Therefore, we present our consecutive single-center case series of recanalized patients with “smaller” MeVOs and DVOs, in which the novel Q catheter was used, providing a granular analysis of procedural success stratified by technique and vessel caliber. By specifically focusing on a cohort of distal occlusions beyond the typical target range of prior mono- and multicenter studies, our study addresses a clinically relevant yet underrepresented subgroup and aims to delineate the incremental value of current endovascular strategies in this more distal vascular territory, while also comprehensively reporting safety and clinical outcomes.

## Material and methods

### Patient data

For our retrospective study, we identified all patients from our stroke center database who underwent thrombectomy of a MeVO or DVO using a MIVI-Q catheter. As standard recanalization technique of LVOs and large MeVOs (according to the discretion of the interventionalist) in our center always includes balloon guiding catheters (BCGs) and the available ones were not compatible with the Q catheters, the collective is highly selective for small MeVOs and DVOs. In these occlusion locations, the use of BCGs was not considered as mandatory, and therefore Q catheters were used as standard approach.

### Definition of MeVO and DVO

**Fig. 1:**
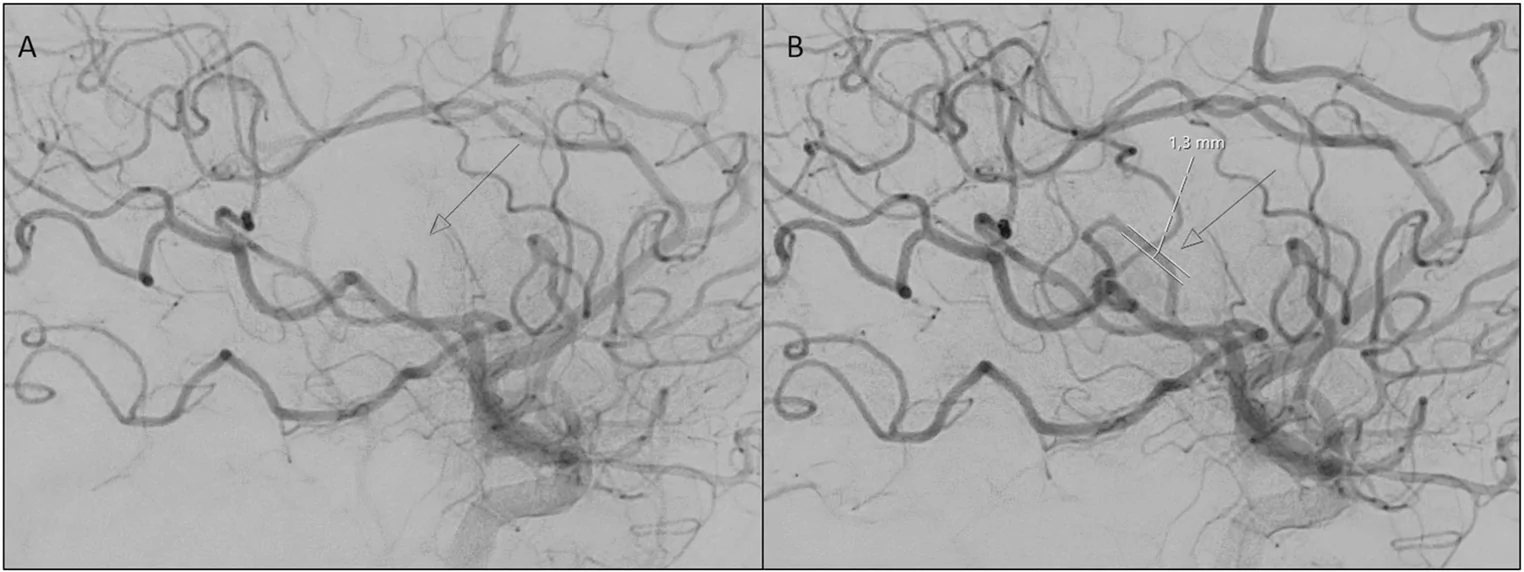
**A**: Preinterventional and **B**: postinterventional image of a left M2 occlusion with a diameter of 1.3 mm. Consequently, classified as DVO

To be precise and comprehensible, occlusions were classified into MeVO and DVO based on the work of Saver et al. [13]. Specifically, M1 occlusions of the middle cerebral artery were categorized as LVO. Dominant M2 occlusions with a vessel diameter > 2.0 mm were excluded from the study as they were classified as LVO. M2 occlusions with a diameter between 1.5 and 2.0 mm were classified as MeVO, whereas M2 occlusions < 1.5 mm were designated as proximal distal vessel occlusions (PDVO) and thus counted as DVO. The DVO group additionally included all M3 and M4 occlusions, A3 occlusions of the anterior cerebral artery, P2 occlusions of the posterior cerebral artery, and superior cerebellar artery (SCA) occlusions. A2 occlusions were classified as MeVO (see Fig. 1).

Vessel diameter was measured postinterventionally in cases of successful recanalization and estimated approximately in cases of failed recanalization.

### Aspiration catheter system

The MIVI Q aspiration catheters (MIVI Neuroscience Inc, Minnesota USA) differ from conventional aspiration catheters according to their design. The proximal three quarters of the MIVI Q catheters consist of a 0.020-inch stainless steel pusher wire which is attached to a catheter resembling the distal part of conventional aspiration catheters. The distal end of this segment is available from 3 F to 6 F and different lengths, accordingly. The proximal part of the aspirational segment is compatible with a guiding catheter with an inner lumen 0.088 inch. Once a vacuum is applied to the guiding catheter it will be transferred to the tip of the MIVI Q catheter, which is meant to significantly increase aspiration force [14].

### Technical and safety outcomes

Technical success was defined as an overall recanalization of mTICI 2b/3, and modified first pass effect (mFPE) as a recanalization success of TICI 2b/3 after one single pass according to the definition of Zaidat et al. [15]. Symptomatic hemorrhage was defined as any hemorrhage with a clinical deterioration of 4 or more NIHSS points or any parenchymal hemorrhage type 2 (PH2) according to the ECASS definition [16]. These definitions were used to allow comparability to the largest published series of small occlusions to date by Alawieh et al. [12].

### Statistics

Descriptive statistics were used to summarize demographic, clinical, and procedural characteristics. Continuous variables are presented as means ± standard deviations (SD), while categorical variables are shown as counts and percentages. Group comparisons between DVO and MeVO were performed using the Mann-Whitney U test for non-normally distributed continuous variables and the independent samples t-test for normally distributed data. Categorical variables were analyzed using the Chi-square test. A p-value < 0.05 was considered statistically significant. All statistical analyses were conducted using Python.

## Results

### Baseline data

A total of 47 patients from 2021 to 2024 were identified, 23 of them females. In 4 patients, multiple vascular occlusions were addressed in a single intervention. In total, 57 vascular occlusions were treated, consisting of 39 DVO and 18 MeVO. One patient was treated twice, and each treatment was counted separately. Mean age of the cohort was 75.5 ± 12.7 and NIHSS on admission was 7.7 ± 4.6. All baseline data are displayed S1 (see supplements 1). The indications to recanalization therapy were based on a predefined in-house SOP (standard of care) guideline, suggesting mechanical thrombectomy in reachable distal vessel occlusions in patients with an NIHSS of ≥ 5 and/or deficits significantly affecting everyday life (e.g. isolated aphasia).

In all patients, Q-catheters were used. In five patients, Q-catheters were employed as a second-line device after failure of the initial approach, resulting in secondary success in four of these five cases. In six cases, after unsuccessful attempts with Q-catheters, an alternative approach was used, leading to secondary success in five of those six cases.

### Technical outcome

Overall, 43 of 57 (75%) could be treated successfully, defined as mTICI (treatment of cerebral ischemia) ≥ 2b. In the following, we further refined our analysis by comparing several different occlusion types and technical approaches.

### Success rates according to technical approach in MeVO and DVO

First, we compared success rates according to technical approach: (1) Pure aspiration and (2) Combined maneuver with aspiration and stent retriever.

In 8 cases, pure aspiration was not successful and it was switched to a combined approach. These cases were attributed to both groups, respectively.

**Fig. 2:**
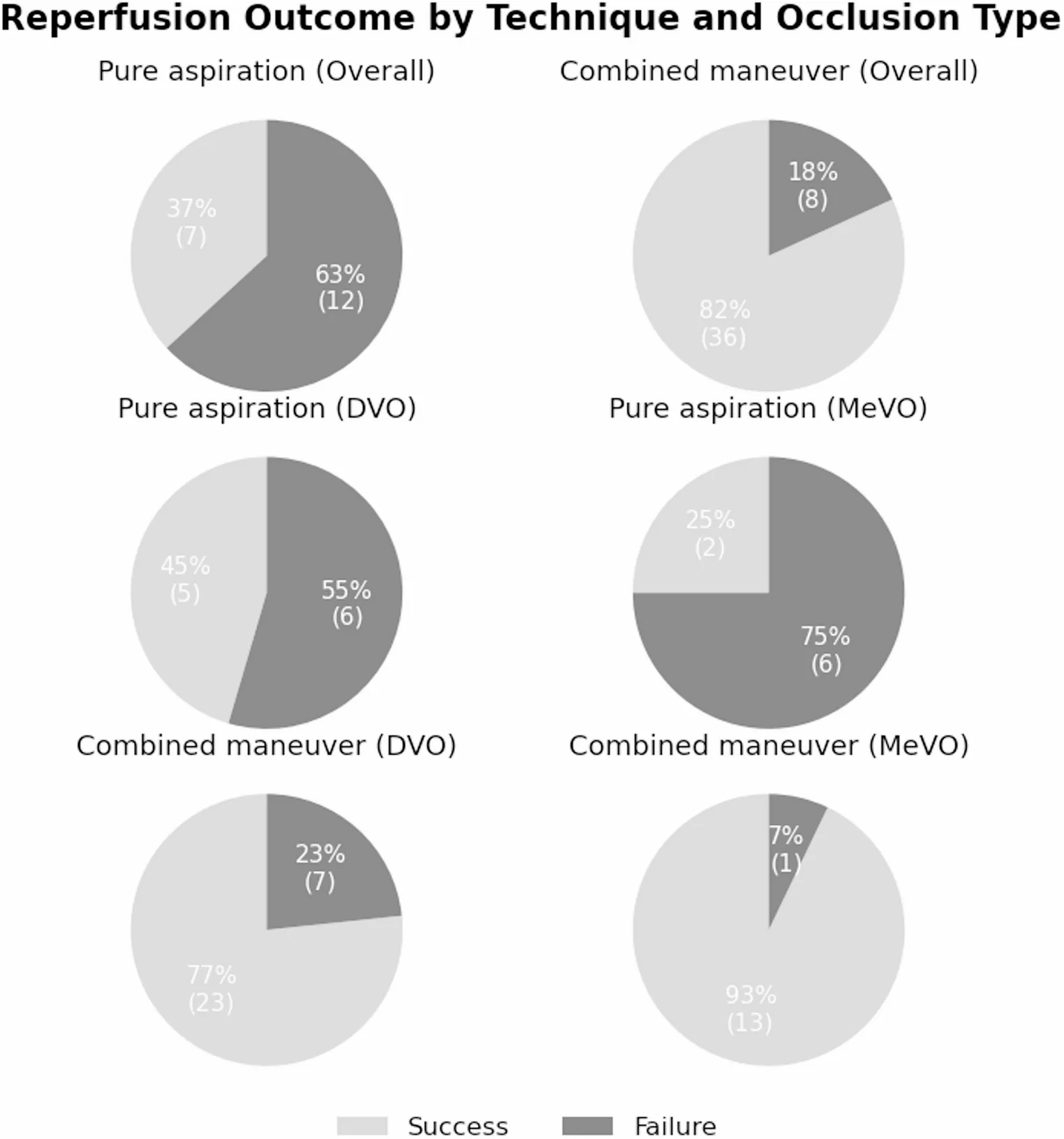
Success rates depending on technical approach overall, in DVO and MeVO

36 of 44 (82%) were successfully recanalized by combined maneuver with aspiration and stent retriever, and just 7 of 19 (37%) with aspiration alone (see Fig. 2). Second, we further divided approaches and success rates according to occlusion location and technical approach: Among the DVOs, 28 of 39 (72%) and among the MeVOs 15 of (83%) could be successfully treated. Combined technique in MeVO in particular showed the highest rate of successful recanalization with 93% (see Fig. 2).

### Success rates in first-line versus second-line use of MIVI Q

In the vast majority of 52 of the 57 cases, the MIVI-Q was used as first line approach, showing a successful recanalization in 40 of our patients. In the remaining 5 cases, the MIVI Q was used as a second-line catheter, meaning that the initial setup for recanalization with another aspiration catheter did not reach successful reperfusion, resulting in a change of strategy towards the MIVI Q. In this case, successful recanalization was achieved in 4 cases.

### Modified first pass effect (mFPE)

In a further analysis, we reviewed how many cases showed complete recanalization after a single thrombectomy maneuver using the mFPE, defined as successful recanalization mTICI ≥ 2b. Here, *n* = 24 (42%) of the *n* = 57 cases showed successful recanalization after a single maneuver.

**Fig. 3:**
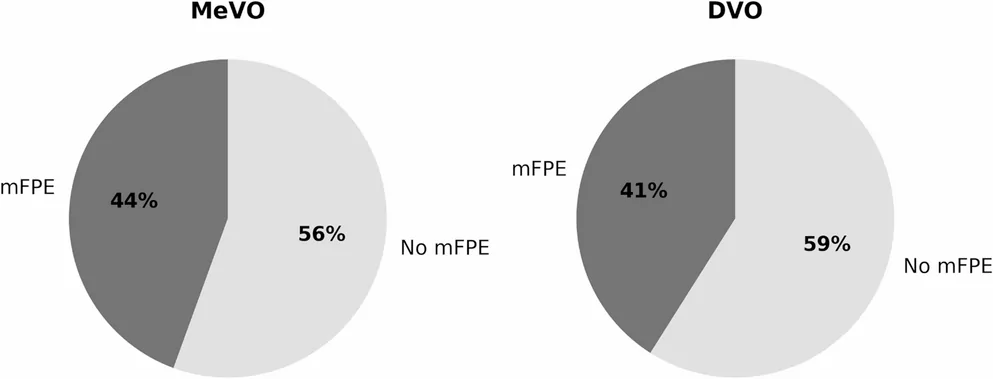
Differentiation of mFPE successes between MeVO and DVO

As shown in S1 and Fig. 3 there was no relevant difference between MeVO (44.4%) and DVO (41%) according to mFPE.

### Safety of Q catheter approach in DVO and MeVO

4 minor or treatable vessel injuries occurred without symptomatic intracranial bleeding as well as one case of ENT (embolization in new territory) leading to an overall complication rate of 9%, none of which with a clear associated clinical deterioration. S1 further details the complications within the various vessels and vessel segments.

It should be noted that 4 of the 5 complications occurred in DVO and, remarkably, 3 of them in A3. Hemorrhage of any type was seen in 25% of cases, mostly consisting of small asymptomatic SAHs.

## Discussion

In this monocentric retrospective study, we analyzed success rates, safety and technical aspects of MIVI Q catheters in the mechanical recanalization of small MeVOs and DVOs in a cohort of 57 vessel occlusions. Overall, 75% of the vessel occlusions in our study were technically successfully recanalized (mTICI ≥ 2b), with 42% showing mFPE. In line with previous studies, successful recanalization rates in MeVOs (83%) were higher compared to DVOs (71%) [12]. Regarding the technical approach, the combined technique with Q catheter and stent retriever showed the highest rates of successful recanalization in MeVOs (93%) and DVOs (77%). Cases initially attempted with aspiration alone but subsequently converted to a combined approach, or vice versa, were included in both groups to accurately reflect all used techniques. Nevertheless, this approach may introduce a substantial bias regarding center-specific preferences; therefore, strict comparisons between techniques should be interpreted with caution.

The main strength of our study is the unique and homogeneous collective of small and very small occlusions. We used the proposed classification of Saver et al. and can present a very large proportion of 39 DVOs and 18 small MeVOs [13]. This large number of very small occlusions is based on the in-house policy of only using non-BGC access (as a prerequisite for the Q-catheter) in initially as very small categorized occlusions obviously lead to a highly selected, but homogeneous patient collective. On the other hand, transferability to other cohorts, which usually encompass a broader spectrum of occlusion types, might be only partially possible.

The mean low NIHHS of 7.7 (7.1 in DEVO and 8.8 in MeVO) in our patients further underlines how small the treated occlusions are compared to other series and are only comparable to the recently published RCT results with 6 in the “Distal” trial and 8 in “Escape-MeVO”. One large retrospective multicenter analysis from the STAR registry seems to contain a similar patient subcollective regarding occlusion locations, but even these patients showed a mean baseline NIHSS of 12 [12]. The only other comparable series to date that published data on with Q recanalized MeVOs consisted of a collective also with an average NIHSS of 12 (in primary MeVOs, *n* = 33) up to 19 (in secondary MeVOs, *n* = 36) [11]. This also underlines the considerable number of patients with small occlusions in our collective.

Despite smaller occlusions, our overall success rate was nearly identical to the RCT results (71.7% in Distal and 75.1% in Escape-MeVO) and also the series of Alawieh et al. with 75%, respectively [12]. In contrast to the other series, our success rate with aspiration alone was comparatively low with 45% in DVO and 25% in MeVO, although the Q catheter is considered to be especially effective by its improved suction force [11, 14]. Nevertheless, in our institute a combined approach is the technical standard and therefore an early switch was probably made very early after the first try with aspiration alone. Therefore, the combined approach turned out to be by far the most successful technique in our local experience (77% in DVOs and 93% in MeVOs).

The, consistent to previous data, lower recanalization rates in DVOs compared to MeVOs is probably multifactorial. Especially after the recent large RCTs, there is no clear evidence for EVT in MeVO and DVO [7, 8]. Therefore, and in anticipation of an expected lower risk-benefit-ratio in cases with low NIHSS and difficult occlusion locations, the interventionalists probably adapted their “aggressiveness” towards a more cautious approach with lower numbers of maneuvers despite persisting occlusions (below 2 in our collective). Overall, the periinterventional complication rate was comparable and acceptable at 9%, particularly since none of these events resulted in clear clinical deterioration. The overall hemorrhage of any kind rate at 25% is in the range of the previously published data by Alawieh et al. [12] 21 (MeVOs) and 22% (DVOs). In our collective, no sICH/PH2 occurred, reflecting a good overall safety profile compared to the previously published rates of between 5% (MeVOs) and 6% (DVOs) [12]. However, the observation that 4 of the 5 complications occurred in DVO and 3 of them in A3 further strengthens a recommendation of a strict review of the respective indication and, if positive, of increasing caution the more distal and smaller the occlusion is located. Especially in the distal segments of the ACA, the complexity of the procedures seems to increase, which is at least in trends also visible in the RCT results. In summary, the use of MIVI Q catheters for mechanical thrombectomy in small MeVOs and DVOs appears to be a feasible and safe approach, achieving recanalization rates comparable to contemporary studies despite the treatment of particularly distal and small vessel occlusions.

## Limitations

The main limitation of this study is its retrospective, single-center design, which inherently carries the risk of selection bias while on the other hand providing a more homogeneous collective. Analyses were performed per occlusion rather than per patient, limiting the interpretation of clinical outcomes, and no comparator group using other aspiration catheters was included, as this was the standard approach in our center.

The institutional policy of using the Q catheter mainly in non-BGC (balloon-guided catheter) cases resulted in a highly selected cohort, introducing a notable selection bias. At the same time, this approach produced a homogeneous and unique collective with a high proportion of very distal and small occlusions—an important strength of this series. Nevertheless, the selective nature of the cohort limits the generalizability of our findings, and observed success rates and technical outcomes should not be directly extrapolated to standard practice, as other centers may employ different catheter strategies or access techniques.

Some procedures initially attempted with aspiration alone were converted early to a combined technique, which further reduces strict comparability between groups. Additionally, case numbers remain limited, as the MIVI Q is a relatively new device. All results were reported by the center itself, albeit re-reviewed by experienced neurointerventionalists, and were not verified by an independent core lab.

## Supplementary Information

Below is the link to the supplementary material.

Supplementary Material 1

## Data Availability

No datasets were generated or analysed during the current study.
