# Correction to: Recanalizing distal and small medium vessel occlusions – single-center experience with the new Q catheters

**DOI:** 10.1007/s00234-026-04127-0

**Published:** 2026-08-03

**Authors:** Michael Griessmair, Christian Maegerlein, Dominik Sepp, Dennis Martin Hedderich, Jannis Bodden, Silke Wunderlich, Claus Zimmer, Jan Kirschke, Maria Berndt-Mück, Martin Renz, Tobias Boeckh-Behrens

**Affiliations:** 1 Department of Neuroradiology, Technical University of Munich, Munich, Germany; 2 Department of Diagnostic, Interventional and Pediatric Radiology, University Hospital of Bern, Bern, Switzerland; 3 Department of Neurology, Technical University of Munich, Munich, Germany

**Correction to: Neuroradiology**

**https://doi.org/10.1007/s00234-026-04026-4**

The original version of this article was revised.

In this article the author’s name Boeckh-Behrens was incorrectly written as Boeck-Behrens, the letter 'h' was missing.

The original article has been corrected.

