# Supplementary material for: Recanalizing distal and small medium vessel occlusions – single-center experience with the new Q catheters
Source: Neuroradiology. 2026 May 9;68(8):2323–9. doi: 10.1007/s00234-026-04026-4 (PMC13578080; doi:10.1007/s00234-026-04026-4)
Supplement: Supplementary file 1 [file 234_2026_4026_MOESM1_ESM.docx]

**Supplements**

| **Characteristic** | **DVO (n=39)** | **MeVO (n=18)** | **Total (n=57)** |
| --- | --- | --- | --- |
| **Sex (m/f)** | – | – | 47 (24 men / 23 women) |
| **Mean age ± SD** | 75.9 ± 12.5 | 78.3 ± 11.8 | 75.5 ± 12.7 |
| **Occlusion location** | M2: 10 (26%) M3: 10 (26%) M4: 2 (5%) P2: 7 (18%) A3: 8 (21%) SUCA: 1 (3%) | M2: 16 (89%)  A1: 1 (6%)  A2: 1 (6%) | DVO: 39 (62%)  MeVO: 18 (38%) |
| **Mean number of maneuvers ± SD** | 1.67 ± 1.08 | 2.00 ± 1.27 | 1.78 ± 1.10 |
| **First-line vs. second-line** | 35 (90%) vs. 4 (10%) | 17 (94%) vs. 1 (6%) | 48 (91%) vs. 5 (9%) |
| **NIHSS at admission (± SD)** | 7.1 ± 4.5 | 8.8 ± 4.6 | 7.7 ± 4.6 (p = 0.21) |
| **NIHSS at discharge (± SD)** | 3.8 ± 4.4 | 6.4 ± 6.8 | 4.85 ± 5.51 (p = 0.21) |
| **mRS after 3 months (Median [IQR])** | – | – | 3 (IQR 4); mRS 0–2 in 37% |
| **mFPE rate** | 16 of 39 (41%) | 8 of 18 (44%) | 24 of 57 (42%) (p = 0.22) |
| **mTICI ≥ 2b** | 28 of 39 (72%) | 15 of 18 (83%) | 43 of 57 (75%) (p = 0.21) |
| **Time from groin puncture to recanalization (≥ TICI 2b)** | 72 ± 40 min | 64 ± 40 min | 67 ± 39 min (p = 0.38) |
| **Complications** |  |  |  |
| **• Any type of hemorrhage** | 10 of 39 (26%) | 4 of 18 (22%) | 14 of 57 (25%) |
| **• PH2 or sICH** | 0 of 39 (0%) | 0 of 18 (0%) | 0 of 57 (0%) |
| **• Vessel injury*** | 3 of 39 (8%)  (2 in ACA A3 segment,1 in MCA M4 segment) | 1 of 18 (6%)  (MCA M2 segment) | 4 of 57 (7%) |
| **• ENT (embolization in new territory) *** | 1 of 39 (3%)  (ACA A3 segment) | 0 of 18 (0%) | 1 of 57 (2%) |

# * Treatment details: Vessel injuries managed with local endovascular treatment without clinical deterioration. Extracranial bleeding was treated conservatively due to diffuse coagulopathy.

Table S1: Baseline characteristics
